# Incidental findings detected with panoramic radiography: prevalence calculated on a sample of 2017 cases treated at a major Italian trauma and cancer centre

**DOI:** 10.1007/s11282-020-00488-1

**Published:** 2020-11-19

**Authors:** Sogol Ghassemzadeh, Luca Sbricoli, Anna Chiara Frigo, Christian Bacci

**Affiliations:** 1 Unit of Oral Medicine, Pathology and Surgery, Section of Clinical Dentistry, Department of Neurosciences, University of Padova, Via Giustiniani, 1, 35128 Padova, Italy; 2 Department of Public Health, University of Padova, Padova, Italy

**Keywords:** Panoramic radiography, Incidental findings, Atherosclerosis, Carotid artery calcification, Elongation of styloid process, Maxillary sinus

## Abstract

**Objectives:**

This study aimed to assess the prevalence of incidental findings, not strictly related to dentistry, viewed with panoramic radiography.

**Methods:**

Panoramic radiographs performed between December 2013 and June 2016 were retrospectively collected. These images were analyzed, searching for incidental findings. All the information collected was statistically analysed

**Results:**

A total of 2307 Panoramic Radiograph were analyzed and 2017 of them were included in the study. 529 incidental findings were seen: 255 (48.2%) were ESP (Elongation of Styloid Process), 167 were CAC (Carotid Artery Calcification) (31.57%), 36 were maxillary sinus pathologies (6.8%) and 71 were other incidental findings (13.42%). The total prevalence of IF was 26, 23%., CAC was 8.28% in the total population, and it was higher in women (9.82%) than men (6.54%). 48.5% of CAC were bilateral. When unilateral, the right side showed a higher right side prevalence. The prevalence of ESP was 12.64% in total population (men: 13.82%; women: 11.60%). 84.71% of ESP were bilateral and, when present unilaterally, no side difference was seen. 13.33% of the ESP appeared segmented. The prevalence of maxillary sinus pathologies was 1.78% (men: 2.32%; women: 1.31%). Only 8.33% of these pathologies were bilateral, and, when unilateral, they were mostly present on the right side. Between the 71 other IF (prevalence: 3.52%), sialoliths and tonsilloliths were assessed most frequently.

**Conclusion:**

Due to the high prevalence of incidental findings detected with panoramic radiography, dental practitioners should be aware of the various pathologic conditions seen on the panoramic radiographs.

## Aim

The present study aimed to estimate the prevalence of incidental findings, detected using Panoramic Radiograph (PR) examinations, with the exception of caries, periodontal and periapical diseases.

Evaluation of PRs, in assessing carotid artery calcification, elongated stylohyoid process, maxillary sinus pathologies, and any other incidental findings, was made.

## Materials and methods

A consecutive case series of panoramic radiographs performed in the University Hospital of Padova between December 2013 and June 2016 were retrospectively collected. The study was performed in full accordance with the World Medical Association Declaration of Helsinki since none of the patients underwent any radiography for this study. All the patients needed panoramic radiographs for dental treatment procedures, trauma, and necessity to start bisphosphonates therapy, to undergo a transplant or cardiac intervention. Therefore, the bias has been reduced because none of these PRs were taken to highlight the anomalies described in the aim of this study.

The panoramic radiographs had been made using the same digital panoramic system. The PRs were examined by 3 expert dentists working in the University of Padova. Finally, a radiologist, who was unaware of the intent of the study, approved all the findings. During the examination of the radiographs, no time limit was imposed.

The PRs were observed in full-screen mode. Overexposed images or calcifications that had low radiopacity were clarified or zoomed in using software tools. If a patient had multiple PRs, only one of them was included in the study, choosing the one with the best image quality. Radiographs that were distorted because of the subjects’ movements during the exposure and those that did not include C3 and C4 vertebrae were excluded. Images with an ambiguous diagnosis due to superimposed structures or lack of clarity, were discarded as well. PRs that did not display the stylohyoid ligament complex in good image quality or in which the origin of the styloid process from the lower part of the temporal bone was hidden by the shadows of the base of the skull, were not included in the study. The presence of radiolucent lesions and radiopacities compatible with mineralization were studied, as well as their unilateral or bilateral characteristics. The patient’s age and sex were recorded for each radiograph reviewed.

- (a) Carotid artery calcifications
  Since their presence was first reported in 1981 [1], radiopacities in the lateral parts of the neck, have received more attention. In fact, in digital panoramic radiographs, the area situated inferiorly and laterally to the hyoid bone, corresponds to the bifurcation of the internal and external carotid arteries, which is exactly where carotid artery calcifications (CAC) are often present. In panoramic radiographs, the bifurcation of the internal and external carotids is situated around the intervertebral discs C3 and C4 or at a 45° to the jaw angle [2, 3]. For this reason, nodular and heterogeneous radiopacities found in these areas may be identified as atherosclerotic calcifications of the carotid arteries. Nonetheless, false-positive results are still possible, because other radiopaque structures are present in these areas, like the triticeous cartilage [4].
  In the present study, irregular and heterogeneous radiopacities, occurring at the bifurcation of the carotid arteries, at the level of C3–C4, adjacent to the cervical spine and hyoid bone, were diagnosed as carotid artery calcifications (CAC).
  The position of every CAC was also recorded: unilateral (right side or left side) or bilateral.
  In all the positive findings, the patients’ age and sex were also assessed.
- (b) Elongated stylohyoid process
  When an elongated stylohyoid process (ESP) was observed, its length was measured, according to the study of Jung et al. [5]. According to his study, the measurements are performed from the frontal view of the styloid process, where it leaves the tympanic plate of the temporal bone. The panoramic radiograph in this area presents a thin transparent line between the styloid process and the tympanic bone, which corresponds to the bone cleft present. The starting point of measurement (A) is situated where this radiolucency fades and the styloid process starts. On the other hand, the ending point of the measurement (B), is the radiopaque tip of the styloid process. The length of the line going from A to B is considered the length of the elongated styloid process.
  The lengths of these mineralized areas were measured using the electronic ruler and recorded.
  Values higher than 25 mm were adopted as the manifestation of an elongated stylohyoid process. The position of the ESP was recorded as well: bilateral or unilateral (right side or left side). In the end, ESPs where broken into two categories: segmented and non-segmented. If no distinction between the stylohyoid ligament complex and the ossified stylohyoid ligament was possible, the entire osseous length of the stylohyoid apparatus was measured as a single unit and was considered non-segmented. Otherwise, if the ESP appeared fragmented, the segments were individually measured and then added together, obtaining the total osseous length.
- (c) Maxillary sinus pathologies
  The maxillary sinus area of the panoramic radiographs was then critically analyzed to identify pathological radiolucencies or radiopaque formations. For all of them, we recorded the position (right side, left side or bilateral), patients’ age and sex.
- (d) Other incidental findings
  The panoramic radiographs included in the study were analyzed to identify all the other entities that did not appear as ‘normal’ and could not be included in the previous categories. All these were considered “other incidental findings”. To correctly diagnose every single entity, differential diagnosis between them was made.

All procedures followed were in accordance with the ethical standards of the responsible committee on human experimentation (institutional and national) and with the Helsinki Declaration of 1975, as revised in 2008.

Informed consent was obtained from all patients for being included in the study, Additional informed consent was obtained from all patients for which identifying information is included in this article.

### Statistical analysis

After data collection, we found the prevalence of patients presenting incidental findings. We then calculated the percentage of female and male patients, and the mean age in every category. We also studied the side distribution of every single finding.

The population was divided into 7 groups, according to the length of the ESP:

**Table:** 

Group	ESP length (mm)
I	≤ 25
II	[25; 30]
III	[30;40]
IV	[40;50]
V	[50;60]
VI	[60;80]
VII	≥ 80

Each group was then further divided into 5 subgroups based on the age: < 20 years; 20–39 years; 40–59 years; 60–79 years; ≥ 80 years. In every single group, the prevalence of female and male population was calculated.

## Results

A total of 2307 panoramic radiographs were collected. According to the inclusion and the exclusion criteria, a final sample of 2017 radiographs was reached, with 1069 females (53%), 948 males (47%) and a ratio of 1:1.13 males to females. These individuals were aged between 6 and 91 years (mean = 47; SD = 16.25) Table 1.

**Fig. 1:**
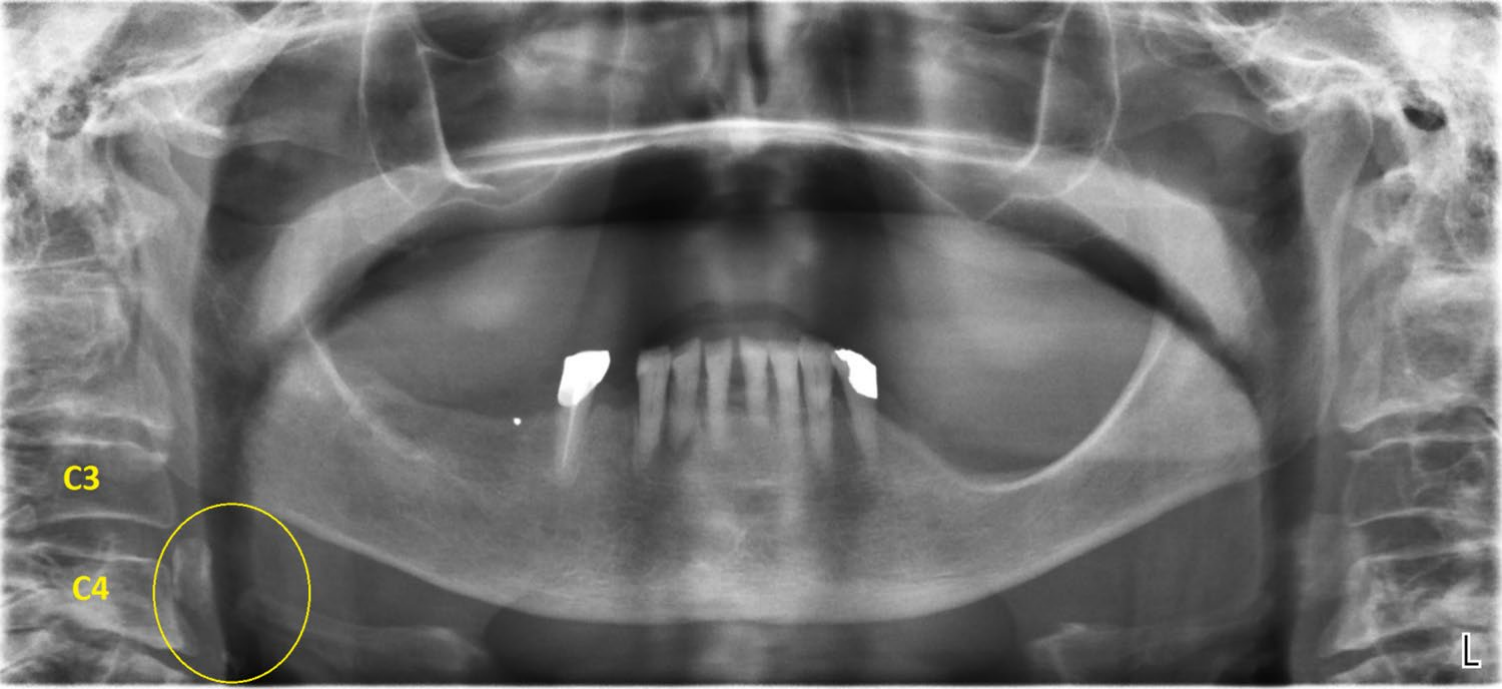
Bilateral carotid artery calcifications at the level of the third cervical vertebra. Superior horn of thyroid cartilage and triticeous cartilage observable at the level of the fourth cervical vertebra

- (a) Carotid artery calcifications
  Of these 2017 subjects, 167 (8.28%) were found to have radiopaque lesions that were identified as CAC.
  The 167 individuals with CAC consisted of 105 females (62.87%) and 62 males (37.13%), with a prevalence of 9.82% in women and 6.54% in men.
  By analyzing the significance of the chi-square test, we observed that gender had a statistically significant association with the presence of CAC. In fact, females presented a greater risk of radiographic presentation of CAC than men Table 2.
  The mean age of these patients was 65.33 years (men: 64.35, women: 65.90). There was no significant difference in mean age between men and women Table 3.
  Of these 167 cases of CAC, 81 (48.5%) were bilateral (52 females, 29 males), 32 (19.16%) were located on the left side (21 females, 11 males) and 54 (32.34%) were located on the right side (32 females, 22 males).
  The prevalence of bilateral CAC was significantly higher than the prevalence of CAC on the right and the left side, for both male and female patients Fig. 1.

**Table 1:** Characteristics of the analyzed population

*n*. Total patients	*n*. males	*n*. females	% Males	% Females	Average age
2017	948	1069	47.00	53.00	47

**Table 2:** CAC prevalence

Carotid Artery Calcification	*n*. Total	Prevalence (%)	*n*. Males	Male prev (%)	*n*. Females	Female prev (%)	Average age
	167	8.28	62	6.54	105	9.82	65.33

**Table 3:** CAC distribution

	Carotid Artery Calcification						
	*n*. Total	Bilateral	Right	Left	Bilateral (%)	Right (%)	Left (%)
Total	167	81	54	32	48.50	32.34	19.16
Males	62	29	22	11	46.78	35.48	17.74
Females	105	52	32	21	49.52	30.48	20

### Elongated stylohyoid process

Of the 2017 radiographs included in the study, 255 (12.64%) displayed visible elongated stylohyoid processes (ESP). A total of 470 ESP were assessed in these 255 panoramic radiographs.

The prevalence of ESP in men was 13.82%, while in women, it amounted to 11.60% Table 4.

**Table 4:** ESP prevalence

	*n.* Total	Prevalence (%)	*n*. Males	Male prev (%)	*n*. Females	Female prev (%)
Elongation of stylohyoid process	255	12.64	131	13.82	124	11.60

Of these 255 subjects presenting ESP, 216 (84.71%) were bilateral, while 20 (7.84%) were on the right and 19 (7.45%) were on the left side.

Of the 216 bilateral ESP, 113 (44.31%) were males and 103 (40.39%) were females.

13 (5.10%) out of the 20 ESP on the right side, were females, while 7 (2.75%) were males. In the end, 11 (4.31%) out of the 19 ESP on the left side were males, and 8 (3.14%) were females.

Out of the 255 cases of ESP, 221 (86.67%) were non-segmented (114 females, 107 males) and only 34 (13.33%) were segmented (24 males, 10 females).

Thus, the prevalence of the non-segmented ESP was significantly higher than the segmented one and the prevalence of bilateral ESP was significantly higher than the ones present on the right and the left side Tables 5 and 6.

**Table 5:** ESP location and type, relating to sex

	Elongation of Stylohyoid Process						
	*n.* Total	Bilateral	Right	Left	Bilateral (%)	Right (%)	Left (%)
Total	255	216	20	19	84.71	7.84	7.45
Males	131	113	7	11	44.31	2.75	4.31
Females	124	103	13	8	40.39	5.10	3.14

**Table 6:** ESP location and type, relating to sex

	Elongation of Stylohyoid Process				
	*n*. Total	Segmented	Non-segmented	Segmented (%)	Non-segmented (%)
Total	255	34	221	13.33	86.67
Males	131	24	107	9.41	41.96
Females	124	10	114	3.92	44.71

Concerning the length of the ESP, values higher than 25 mm were adopted as the manifestation of the elongated stylohyoid process. In particular, it should be noted that 255 PRs showed ESPs, but the total number of ESPs was 470, given that some of them were unilateral, while others were bilateral.

Out of the identified 470 ESPs as many as 71.28% were found to have a pathological length, i.e. greater than 25 mm.

28.72%, on the other hand had a length ≤ 25 mm.

In the panoramic radiographs with pathological ESP length (> 25 mm), the highest prevalence was found in the 30–40 mm-long group (29.57%), followed by the 25–30 mm-long group (20.64%).

**Fig. 2:**
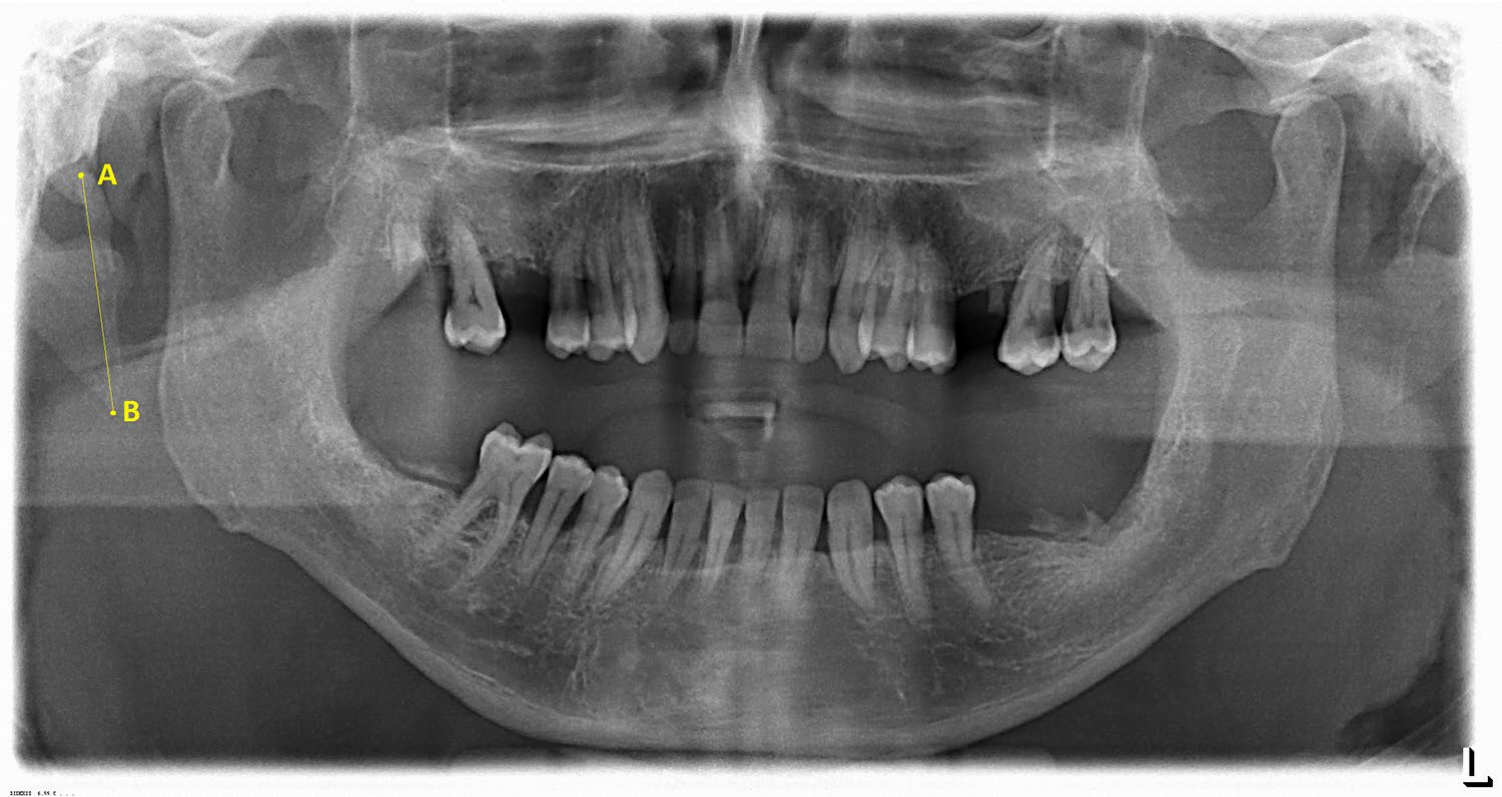
ESP measurement

There was a greater tendency for the abnormality (ESP > 25 mm) in over 40 years old and male patients Table 7; Fig. 2.

**Table 7:** ESP length relating to age and sex

		** < 20 years**				***20–39 years***				***40–59 years***				***60–79 years***				** ≥ 80 years**				***Total***					
		*F*		*M*		*F*		*M*		*F*		*M*		*F*		*M*		*F*		*M*		*F*		*M*			
*Group*	***Length (mm)***	*R*	*L*	*R*	*L*	*R*	*L*	*R*	*L*	*R*	*L*	*R*	*L*	*R*	*L*	*R*	*L*	*R*	*L*	*R*	*L*	*R*	*L*	*R*	*L*	***Total***	***%***
*I*	* ≤ 25*	1	1	0	0	12	6	5	3	17	18	10	10	12	11	15	11	1	0	1	1	43	36	31	25	**135**	28.72
*II*	*[25; 30]*	0	0	0	0	7	9	2	2	6	6	10	10	15	9	6	9	1	3	1	1	29	27	19	22	**97**	20.64
*III*	*[30;40]*	0	0	0	0	3	4	7	10	11	13	13	17	11	14	15	13	2	1	2	3	27	32	37	43	**139**	29.57
*IV*	*[40;50]*	0	0	0	0	2	5	3	3	6	2	13	9	5	4	7	10	1	2	2	2	14	13	25	24	**76**	16.17
*V*	*[50;60]*	0	0	0	0	1	1	1	0	0	0	3	5	0	1	2	3	2	1	0	1	3	3	6	9	**21**	4.47
*VI*	*[60;80]*	0	0	0	0	0	0	0	0	0	0	0	0	0	0	0	0	0	0	1	0	0	0	1	0	**1**	0.21
*VII*	≥ *80*	0	0	0	0	0	0	0	0	0	0	0	0	0	0	0	0	0	0	1	0	0	0	1	0	**1**	0.21
	***Total***	1	1	0	0	25	25	18	18	40	39	49	51	43	39	45	46	7	7	8	8	116	111	120	123	**470**	100

### Maxillary sinus pathologies

Out of the 2017 PRs reviewed, 36 (1.78%) displayed maxillary sinus pathologies: 22 men (61.11%) and 14 women (38.89%). Thus, we obtained a prevalence of 2.32% in men and 1.31% in women Table 8.

**Table 8:** Prevalence of maxillary sinus pathologies

	*n*. Total	Prevalence (%)	*n*. Males	Male pev (%)	*n*. Females	Female prev (%)
Maxillary sinus pathology	36	1.78	22	2.32	1.4	1.31

The mean age of the patients presenting maxillary sinus pathology was 49.64 years, ranging from 11 to 81 years Table 9.

**Table 9:** Maxillary sinus pathologies relating to sex and location

	Maxillary sinus pathologies									
	*n*. Total		Bilateral			Right			Left	
		Total	Females	Males	Total	Females	Males	Total	Females	Males
Sinusitis	22	3	1	2	11	5	6	8	3	5
Antral pseudocist	14	0	0	0	13	5	8	1	0	1
Total	36	3	1	2	24	10	14	9	3	6

The major part of these findings (91.67%) was unilateral, while just 3 (8.33%) were bilateral (see Tables 10, 11).

**Table 10:** Prevalence of other incidental findings

	*n*. Total	Prevalence (%)	*n*. Males	Male prev (%)	*n*. Females	Female prev (%)	Average age
Other incidental findings	71	3.52	45	4.75	26	2.43	54.17

**Table 11:** Total prevalence and single prevalences of incidental findings

Incidental findings	Number	Prevalence (%)
Total	529	26.23
Carotid artery calcification	167	8.28
Elongation of stylohyoid process	255	12.64
Maxillary sinus pathology	36	1.70
Other incidental findings	71	3.52

### Other incidental findings

A total number of 529 incidental findings were seen: 255 (48.2%) were ESP, 167 were CAC (31.57%), 36 were maxillary sinus pathologies (6.8%) and 71 “other incidental findings” (13.42%).

These included: tonsilloliths, sialoliths, residual cysts, radiolucent cyst-like lesions, radiopaque lesions, mixed radiopaque-radiolucent lesions, hyperostosis, foreign bodies of endodontic origin.

Hence, the prevalence of these other kinds of incidental findings was 3.52%.

Of these 71 patients, 45 (63.38%) were men and 26 (36.62%) were women, with a mean age of 54.17 years, ranging from 19 to 85 years.

The “other incidental findings” with the highest prevalence of occurrence were sialoliths and tonsilloliths, amounting, respectively, to 18 cases (25.35%) and 15 cases (21.13%). Hence, the prevalences of sialoliths and tonsilloliths were, respectively, 0.89% and 0.74%.

We then found: 4 residual cysts (2 on the mandibular left side, 1 on the mandibular right side, 1 on the maxillary right side); 3 radiolucent cyst-like lesions (1 on the mandibular right side, 1 in the mandibular symphysis, 1 on the maxillary right side); 23 radiopaque lesions (1 on the maxillary left side and the other 22 occurring in the mandible: 9 on the left side, 7 on the right side, 6 in parasymphysis); 3 mixed radiopaque-radiolucent lesions (2 on the mandibular left side and 1 on the maxillary right side); 4 hyperostosis (3 occurring on the mandibular right side and 1 occurring in the mandibular left side); 2 small radiopaque lesions in the maxillary sinus, which appeared similar to foreign bodies of endodontic origin.

In conclusion, a total number of 529 incidental findings were seen: 48.2% of them were ESP, 31.57% were CAC, 6.8% were maxillary sinus pathologies and 13.42% were other incidental findings.

**Fig. 3:**
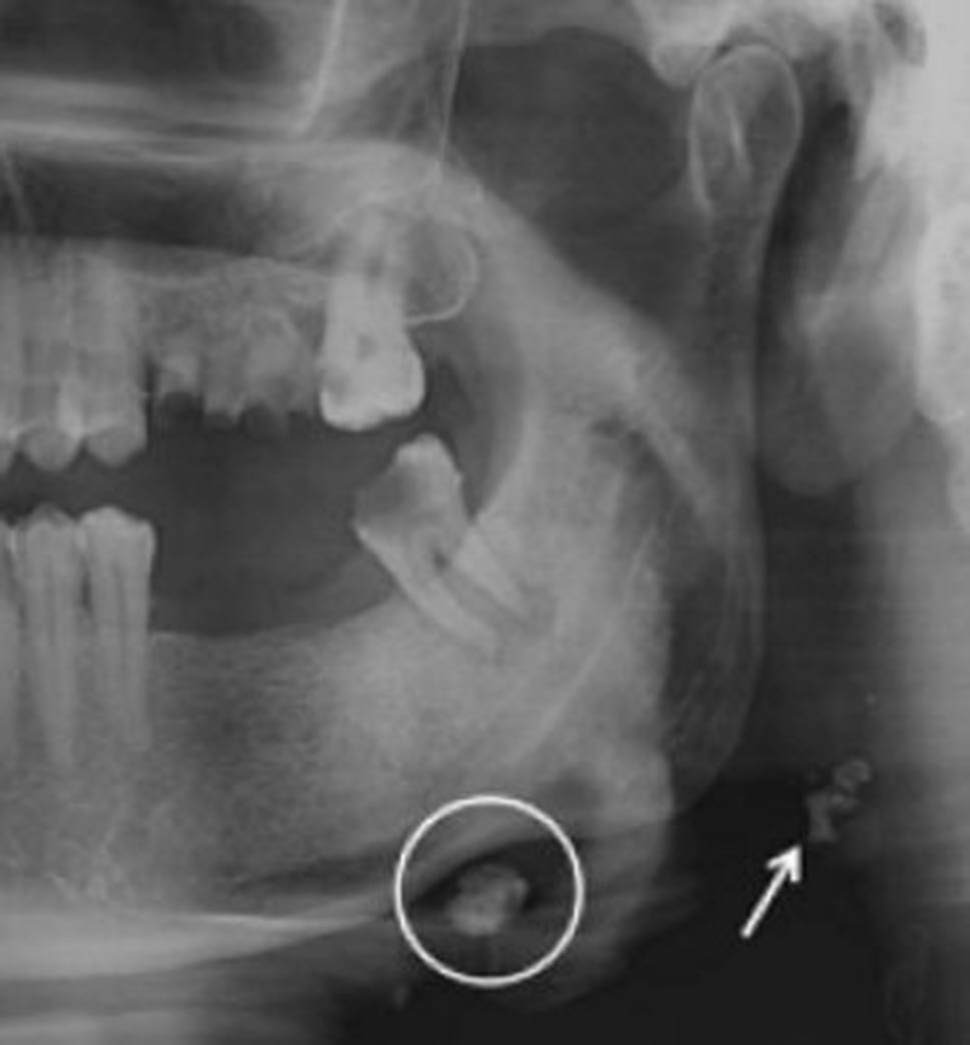
A well-defined, solitary nodular radiopaque structure was seen inferior to the left mandibular border, near the angle. Apart from this, multiple punctuate radiopacities were seen at the level of the lower margin of the cervical vertebra on the left side. Based on the radiographic location and appearance of the lesion on the radiograph, a provisional diagnosis of the left submandibular gland sialolothiasis and carotid artery calcification was made

The total prevalence of the incidental findings was 26,23% Table 12; Fig. 3.

**Table 12:** Percentage of the incidental findings

Incidental findings	Number	Percentage (%)
Carotid artery calcification	167	31.57
Elongation of stylohyoid process	255	48.20
Maxillary sinus pathology	36	6.80
Other incidental findings	71	13.42

## Discussion

A peculiarity of this study was that panoramic radiographs were analyzed for all kinds of incidental findings not related to dentistry. Both the single prevalence of each of them and the overall prevalence were thus obtained.

Also, MacDonald et al. [6] analyzed consecutive PRs of symptom-free patients, but these ones attended a Canadian general dental practice and solely for dental examination or hygiene. Therefore, this sample was not representative of the general population.

### Carotid artery calcification

Cerebral vascular accidents represent the third cause of death in industrialized countries [2]. They are one of the major public health problems, because of their high incidence and the rehabilitation cost of patients [3].

This study showed that panoramic radiography could help to diagnose carotid artery calcifications.

**Fig. 4:**
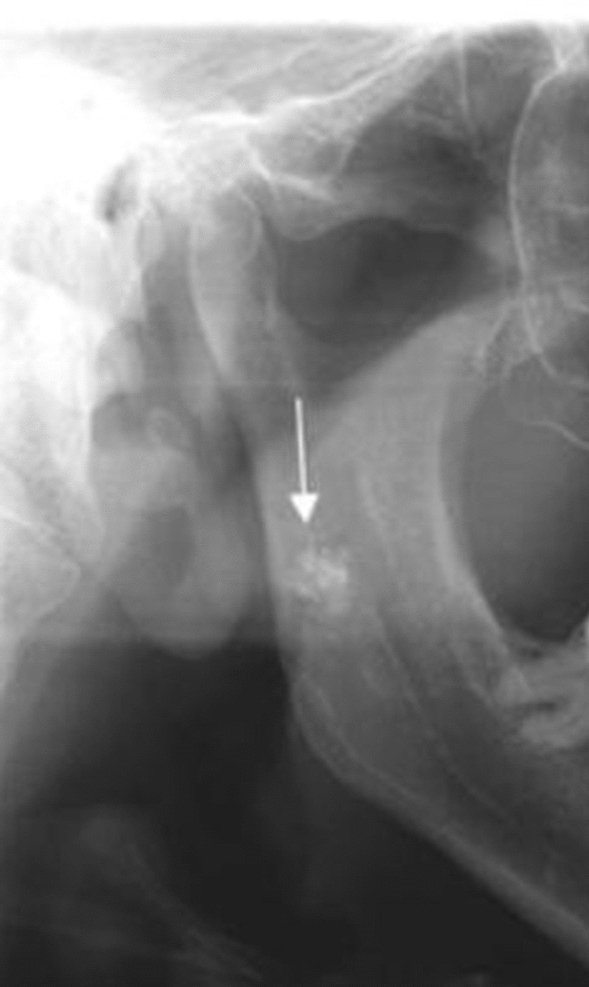
Tonsilloliths are superimposed in the right mandibular ramus

**Fig. 5:**
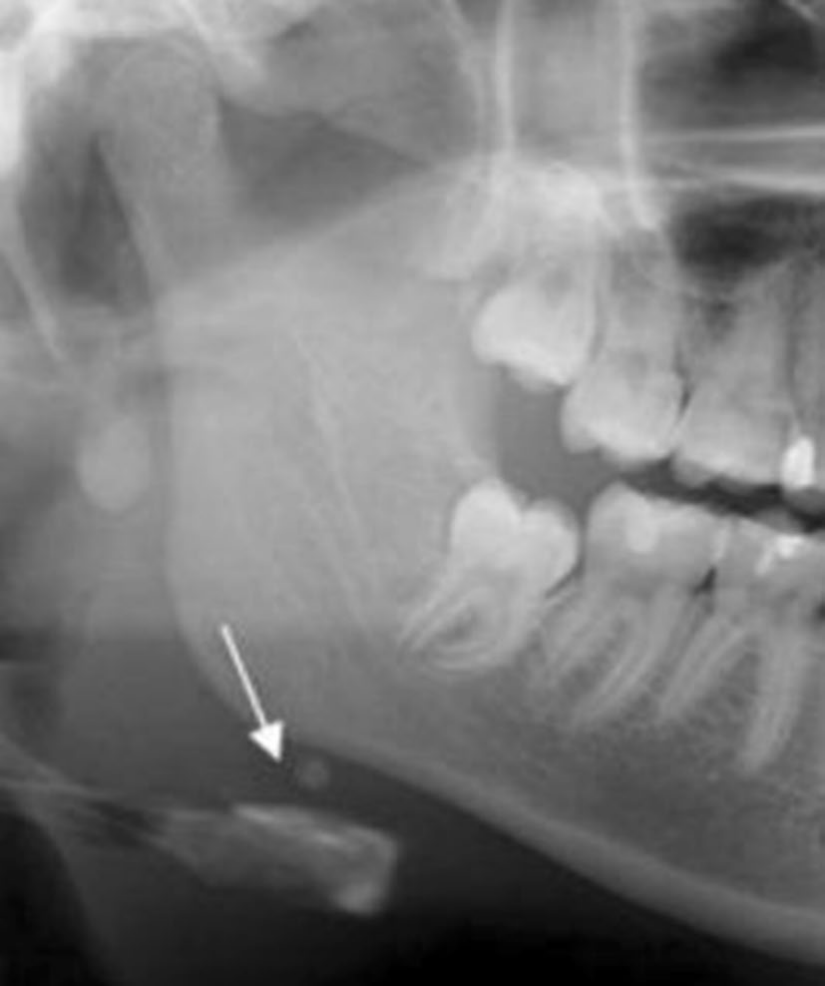
A sialolith under right mandibular border

**Fig. 6:**
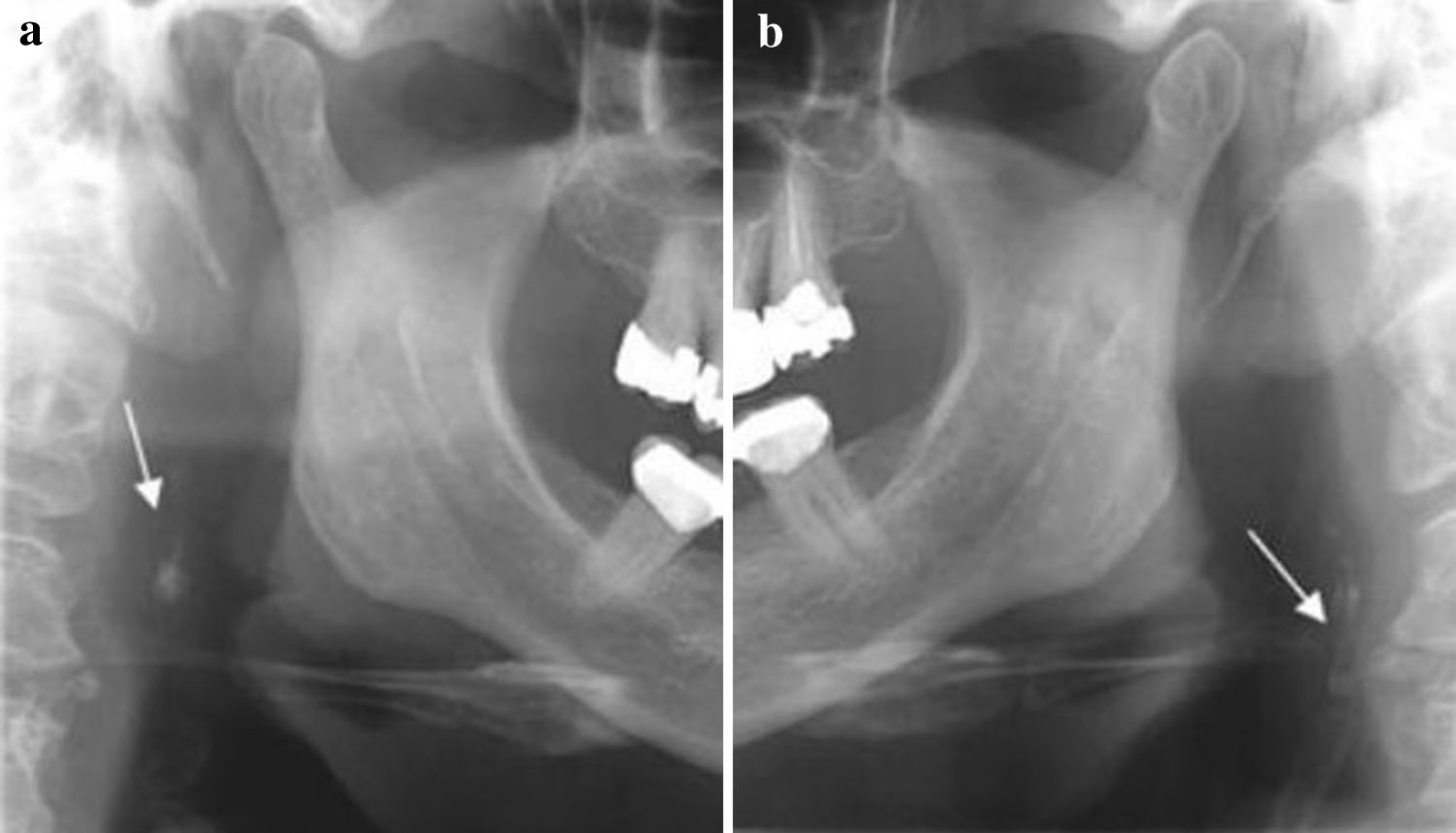
**a**, **b** Bilateral carotid artery calcifications at the level of the third cervical vertebra. Superior horn of thyroid cartilage and triticeous cartilage observable at the level of the fourth cervical vertebra

**Fig. 7:**
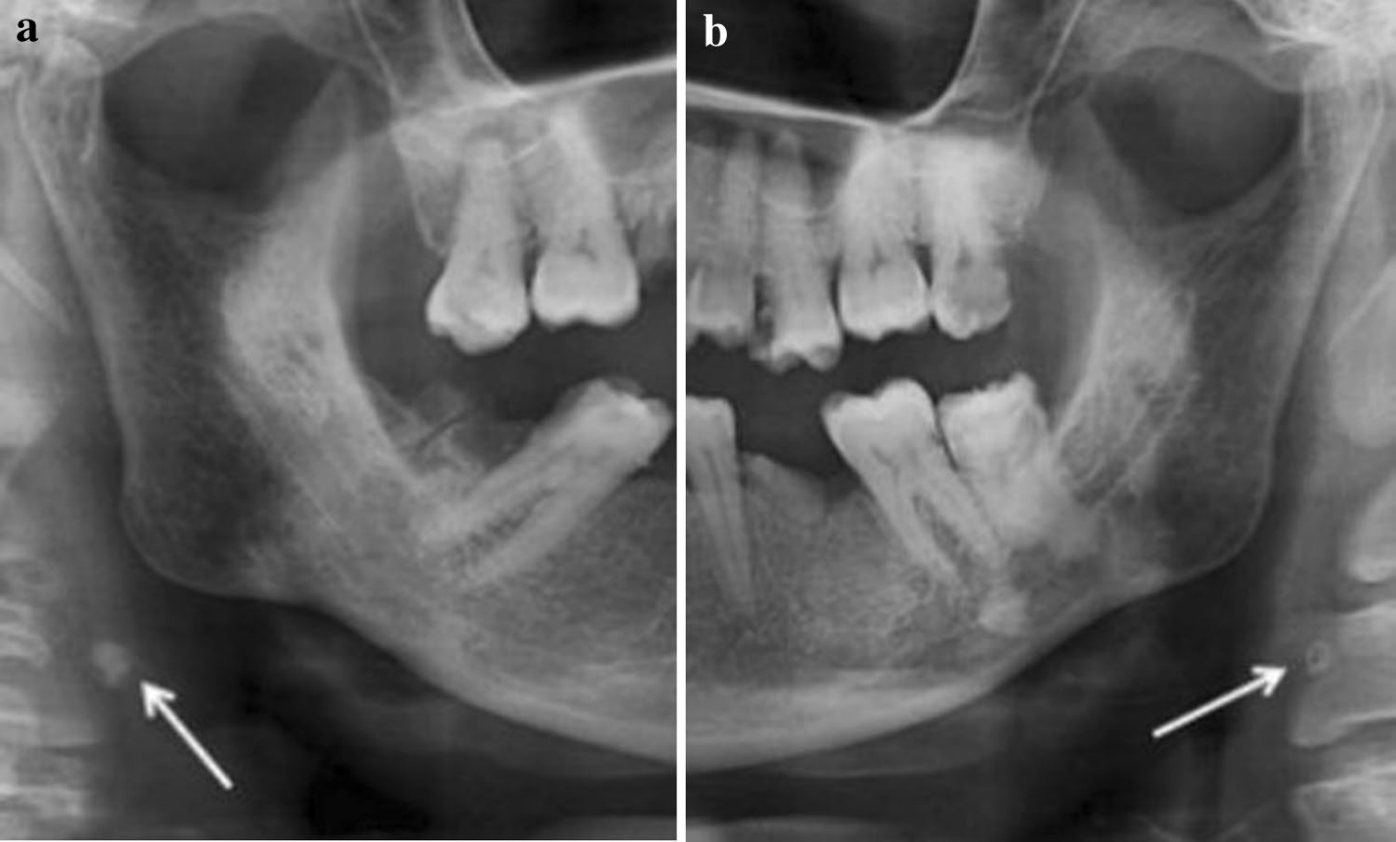
**a**, **b** Well defined, bilateral nodular radiopacities were seen at the level of the lower margin of the third and the fourth cervical vertebra (C3 & C4). Based on the radiographic location and appearance of the lesion on the radiograph, a provisional diagnosis of the coronary artery calcification was made

**Fig. 8:**
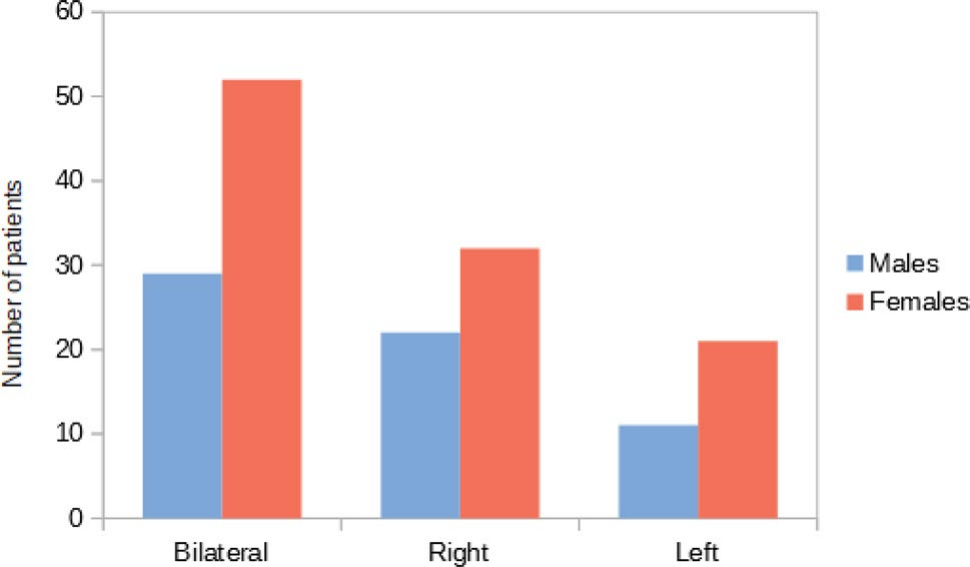
CAC location relating to sex

**Fig. 9:**
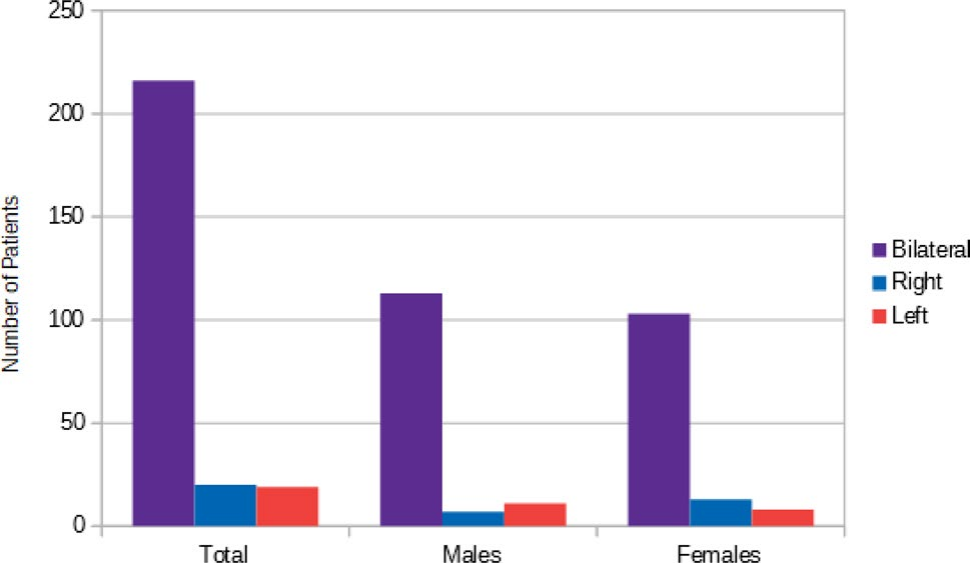
ESP location

**Fig. 10:**
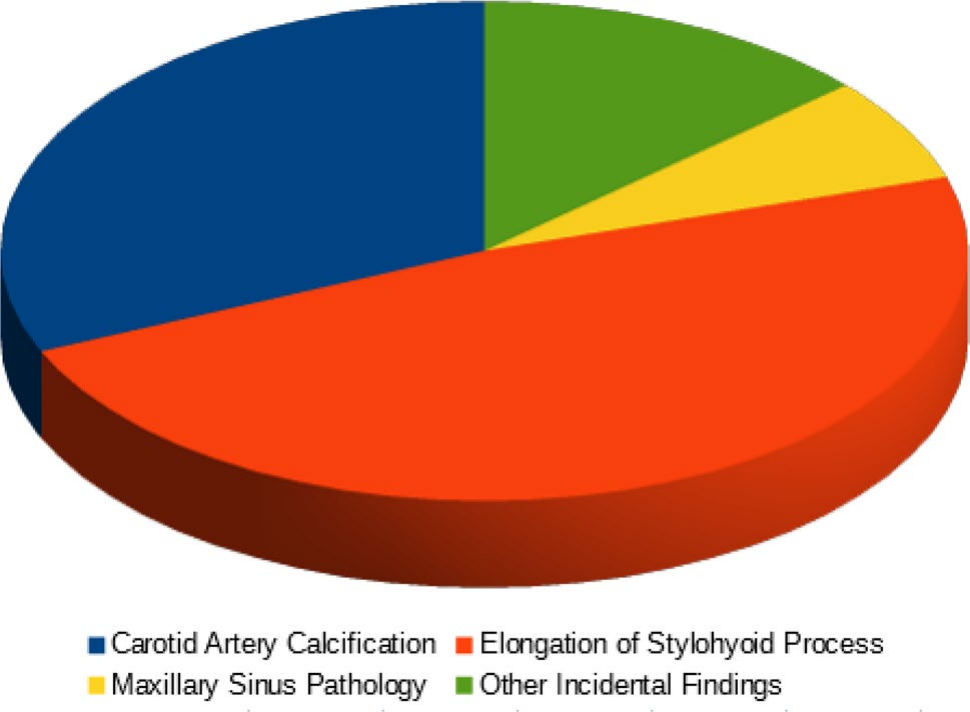
Percentage of the incidental findings

An accurate analysis of PRs for carotid calcifications could be a useful tool in assessing individuals at risk for stroke, although it requires training to detect calcifications on panoramic radiographs Figs. 4, 5, 6, 7, 8, 9 and 10.

In literature, we can find some variance in the prevalence of CAC visible on the PRs.

This study found a prevalence of 8.28% of the presence of carotid artery calcifications on the panoramic radiographs, while Monsour [7] found calcifications in 4% of a total of 2628 non-digital panoramic radiographs. However, our study, unlike the study by Monsour [7], uses digital panoramic radiographs, which enables low-density calcifications to be identified, also because the image contrast can be modified.

Dorado et al. [8] found a prevalence of 15.5%, while Bryam et al. [9] obtained a result of 2.1%.

Monteiro et al. [10] found a prevalence of 9.5%, which is similar to the one found in the present study.

We detected CACs through panoramic radiography in 8.28% of the subjects (with an average age of 65.33 years), and most of them were women (62.87%).

The result of women having a significantly higher prevalence of CAC (9.82% vs a prevalence of 6.54% in men), may suggest a relationship between the decline of estrogen levels in the blood of postmenopausal women and CAC, as proposed by Friedlander and Altoman [11]. In fact, they reported that estrogen decreases low-density lipoprotein (LDL) catabolism in blood and that increased LDL cholesterol levels in blood were associated with the risk of cardiovascular pathologies. Although some studies have suggested an increased prevalence in women, this statistical significance has been contested (Levy and Mandel, [12]).

The recognition of CAC on PR has some limitations, such as quantifying the degree of stenosis. Consequently, Doppler ultrasound is still the main diagnostic test of this pathology, being accurate and non-invasive [13, 14].

### Elongated stylohyoid process

The measurement of the stylohyoid process on a panoramic radiograph was challenged. In fact, a 12 pattern classification, which displayed significant difference between prevalences of different patterns varied between East Asian and Western communities [15].

In our study, the prevalence of ESP was 12.64%. This value is lower than that found by Rizzatti-Barbosa et al. [16].

Several studies have concluded that there is a large variation in the length of the styloid ligament complex, and this varies from individual to individual. In general, styloid processes > 25 mm are considered to be of abnormal length.

The results from the present study indicated that the length of 28.72% of the calcified stylohyoid processes that were found, was shorter than 25 mm, while the remaining 71.28% was longer than 25 mm.

In the panoramic radiographs with pathological ESP length (> 25 mm), the highest prevalence was found in over 40 years old and male patients.

Concerning the spatial distribution of ESPs, our study, just like that of Rizzatti-Barbosa et al. [16], has shown that it could tend to their bilateral position.

Ultimately, the prevalence of the non-segmented ESPs was significantly higher than the segmented ones and the prevalence of bilateral ESPs was significantly higher than the ones present on the right and the left side.

In conclusion, as proposed by Steinmann [17], there are 3 theories to explain ossification of the stylohyoid process: the theory of *reactive hyperplasia,* the theory of *reactive metaplasia,* the theory of *anatomic variation.*

The latter involves the stylohyoid ligament or the styloid process as ossified structures that develop in the early formative years after birth. This theory may fit in radiographic findings of ossification in children and young adolescents [18]. However, our study showed that just 2 out of the 255 panoramic radiographs presenting ESP, were found in patients younger than 20 years old. Hence, this finding may demonstrate that the theory of anatomic variation proposed by Steinman [17] is not valid in our study.

### Maxillary sinus pathologies

Although panoramic radiography is not considered the gold standard for the diagnosis of maxillary sinus pathologies and cannot be used to entirely exclude their presence, it can help to detect some of them. Being frequently used, it may help to identify particularly the asymptomatic ones or the most insidious ones. This may be crucial in the case of malignancies, because early identification can be very important for the prognosis. As reported in literature, early detection can result in at least 80% treatment success rate as determined by the 5-year survival [19, 20]. It has also been found that at the time of diagnosis of antral malignancies, these can be seen on panoramic radiographs in 90% of the cases [21].

Halstead et al. [22], reported that sinus pathologies detected with PR had a prevalence of about 2%. Our study found a similar result, since 1.78% of the radiographs reviewed displayed maxillary sinus pathologies, indicating that PR may be a useful additional tool to detect maxillary sinus diseases. The mean age of these patients was 49, 64 years and they were mostly men (61.11%).

Concerning the spatial distribution, 91.67% of these pathological findings were unilateral, indicating that maxillary sinus diseases are most frequently present on a single side. In particular, the right side showed a higher incidence.

Lastly, antral pseudocyst and maxillary sinusitis showed the highest prevalence, respectively, representing 36.11% and 30.56% of the maxillary sinus pathologies.

The literature reports that the prevalence of mucosal thickening from an inflammatory origin (such as antral pseudocyst and true sinus mucocele), averages around 38.89%, but varies considerably from report to report, perhaps as a function of population, geography and season [22, 23].

Our study included panoramic radiographs performed between December 2013 and June 2016. Hence, all the seasons were included, allowing us to collect a more homogeneous data set.

Compatibly with the literature [22–24], our study found a similar prevalence of maxillary sinus inflammatory diseases, since 38.78% of the sinus pathologies found, were assessed as antral pseudocysts, while the remaining cases appeared as maxillary sinusitis.

### Other incidental findings

The other incidental findings encountered included: sialoliths, tonsilloliths, residual cysts, radiolucent cyst-like lesions, radiopaque lesions, mixed radiopaque-radiolucent lesions, hyperostosis and foreign bodies of endodontic origin.

The prevalence of these occasional findings was 3.52% and the most frequently seen ones were sialoliths and tonsilloliths, with a prevalence of 0.89% and 0.74%, respectively.

According to Mandel [25], tonsilloliths affect male and female patients equally, while Suarez-Cunqueiro et al. [26] described a greater occurrence in adult males (1.6:1).

Our study, congruently to Suarez-Cunqueiro et al. [26], found a higher prevalence in male patients (ratio = 1.25:1).

## Conclusion

This study demonstrated the usefulness of panoramic radiography in showing a broad range of different pathological conditions not strictly related to dentistry, such as carotid artery calcifications, elongated stylohyoid processes, maxillary sinusitis, antral pseudocysts, sialoliths, tonsilloliths, etc. Due to the high prevalence of occasional findings, dental practitioners should be aware of the various conditions that may be encountered.

In particular, the widespread recognition that calcifications seen in the region of the carotid bifurcation can identify a population with a higher risk of stroke, supports the practice of routinely examining this area.

Since a panoramic radiograph is usually performed for dental reasons, an in-depth examination of the image is substantially cost-free and can help to identify unknown pathologies and, thus, prolong lives and bring significant savings in overall healthcare costs.

Therefore, dentists should be able to recognize the widest range of occasional findings that could be found in the PRs, to allow early medical interventions.
